# Ileo-Ileal Knotting: A Rare Cause of Strangulated Small Bowel Obstruction

**DOI:** 10.7759/cureus.89019

**Published:** 2025-07-29

**Authors:** Mohamed Alatrash, Aya M Alamrawy

**Affiliations:** 1 General Surgery, Maidstone and Tunbridge Wells NHS Trust, Royal Tunbridge Wells, GBR; 2 General and Colorectal Surgery, Cairo University, Giza, EGY; 3 General Surgery, Cairo University, Giza, EGY

**Keywords:** acute surgical emergency, ileo-ileal knotting, small-bowel obstruction, surgical case reports, urgent laparotomy

## Abstract

Small bowel obstruction is a common surgical emergency, but ileo-ileal knotting is an exceptionally rare cause in which two loops of ileum intertwine into a true knot, leading to strangulation. Preoperative diagnosis of ileo-ileal knotting is extremely challenging due to nonspecific clinical features, and most cases are identified only during emergency surgery. We present three cases of ileo-ileal knotting to illustrate varied precipitating factors, presentations, and diagnostic challenges associated with this condition. All patients underwent urgent exploratory laparotomy with resection of the knotted, nonviable bowel and primary anastomosis; each had an uneventful recovery and was discharged in good condition. Although rare, ileo-ileal knotting should be considered in the differential diagnosis of acute intestinal obstruction, especially in rapidly deteriorating patients with signs of strangulation, since prompt surgical intervention is essential to prevent a fatal outcome.

## Introduction

Intestinal obstruction represents one of the most common surgical emergencies, necessitating prompt evaluation and timely intervention. Small bowel obstruction, in particular, remains a leading cause of hospital admissions worldwide. Its differential diagnosis is broad and includes postoperative adhesions, neoplasms, hernias, Crohn’s disease, and complications related to prior abdominal or pelvic irradiation [1].

An intestinal knot is a very rare cause of bowel obstruction. It was first described by Riverius in the 16th century and by Rokitansky in 1836 [2].

Several types of intestinal knots have been described, including appendico-ileal, ileocecal, ileo-ileal, ceco-sigmoid, and ileo-sigmoid varieties. Among these, the ileo-sigmoid knot is the most common, while the ileo-ileal knot is considered the rarest [3].

Because of its rarity and lack of specific clinical or radiologic signs, ileal knotting is typically diagnosed intraoperatively during emergency surgery. The condition necessitates urgent intervention, as the vascular compromise of knotted bowel can rapidly progress to intestinal gangrene. We present a case series of three patients with ileo-ileal knotting, each with a different precipitating factor for the knot formation, managed at our tertiary care hospital.

## Case presentation

Case 1

Patient Information

A 50-year-old male with no significant past medical history (no diabetes, hypertension, or prior surgeries) presented to the emergency department.

Clinical Presentation

He had 48 hours of acute, diffuse abdominal pain with bilious vomiting and no bowel movement, followed by an episode of diarrhoea with melena. On examination, he appeared distressed and tachycardic (heart rate ~130/min). His abdomen was rigid with diffuse tenderness and rebound tenderness, indicating peritonitis. A nasogastric tube was inserted, and the output was coffee-ground aspirate.

Diagnostic Assessment

Laboratory studies showed leucocytosis (white blood cell (WBC) count ~23,000/µL). An upright abdominal X-ray revealed multiple air-fluid levels consistent with small bowel obstruction. Given the peritoneal signs, an immediate exploratory laparotomy was indicated without time for advanced imaging.

Therapeutic Intervention

**Figure 1:**
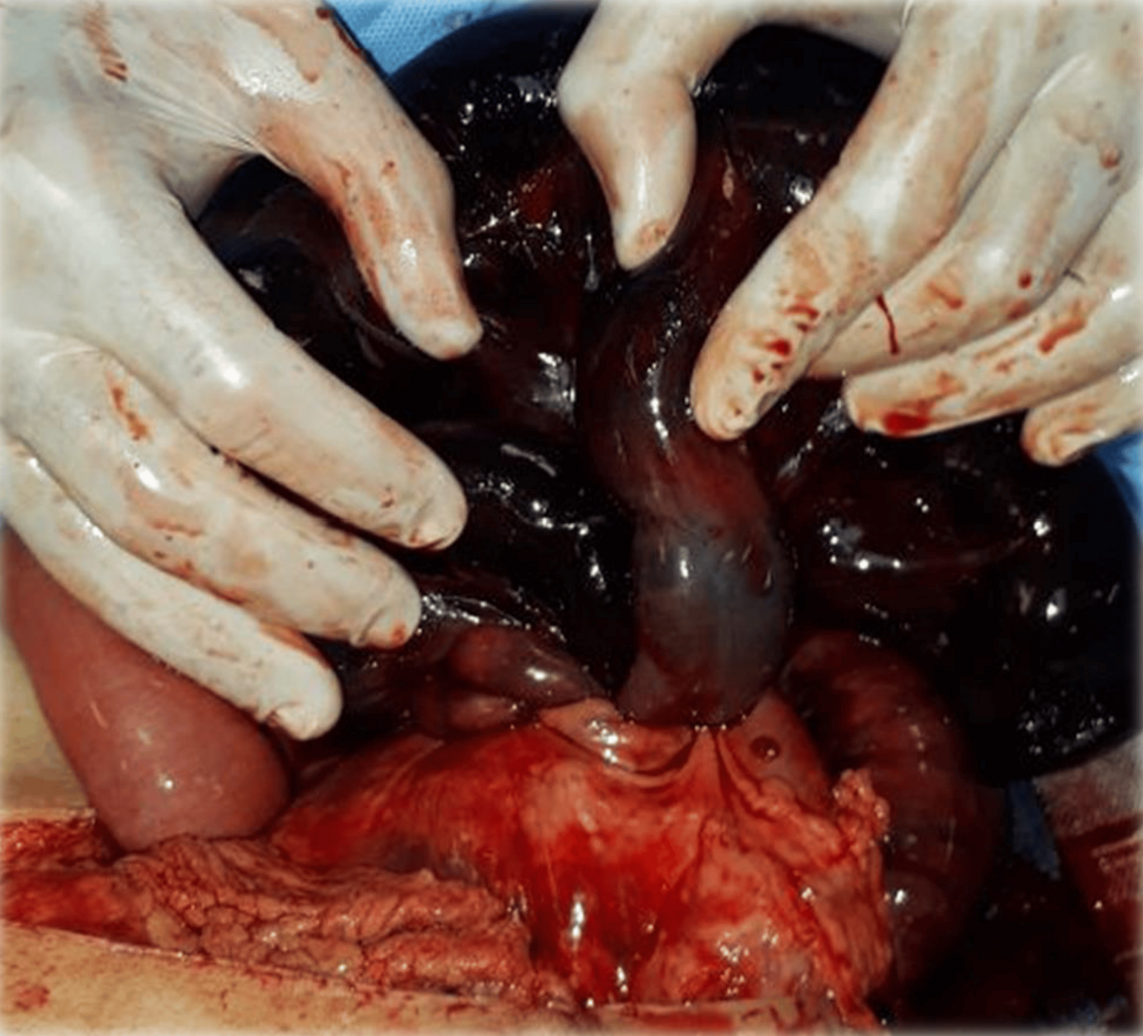
Case 1: Ileal-ileal knotting at the terminal ileum, showing gangrenous small bowel.

**Figure 2:**
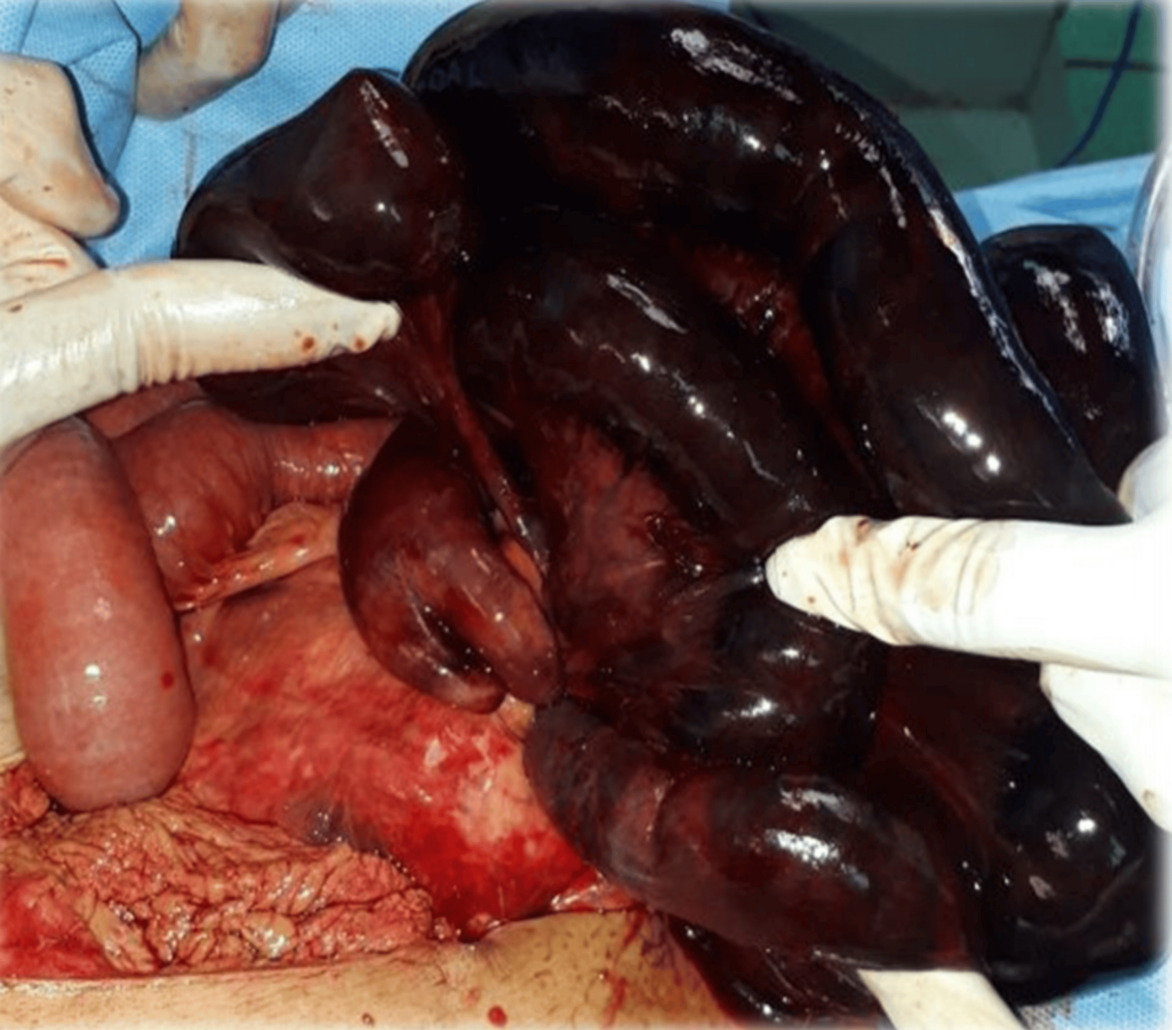
Case 1: Intraoperative image of the knotted small bowel loops.

**Figure 3:**
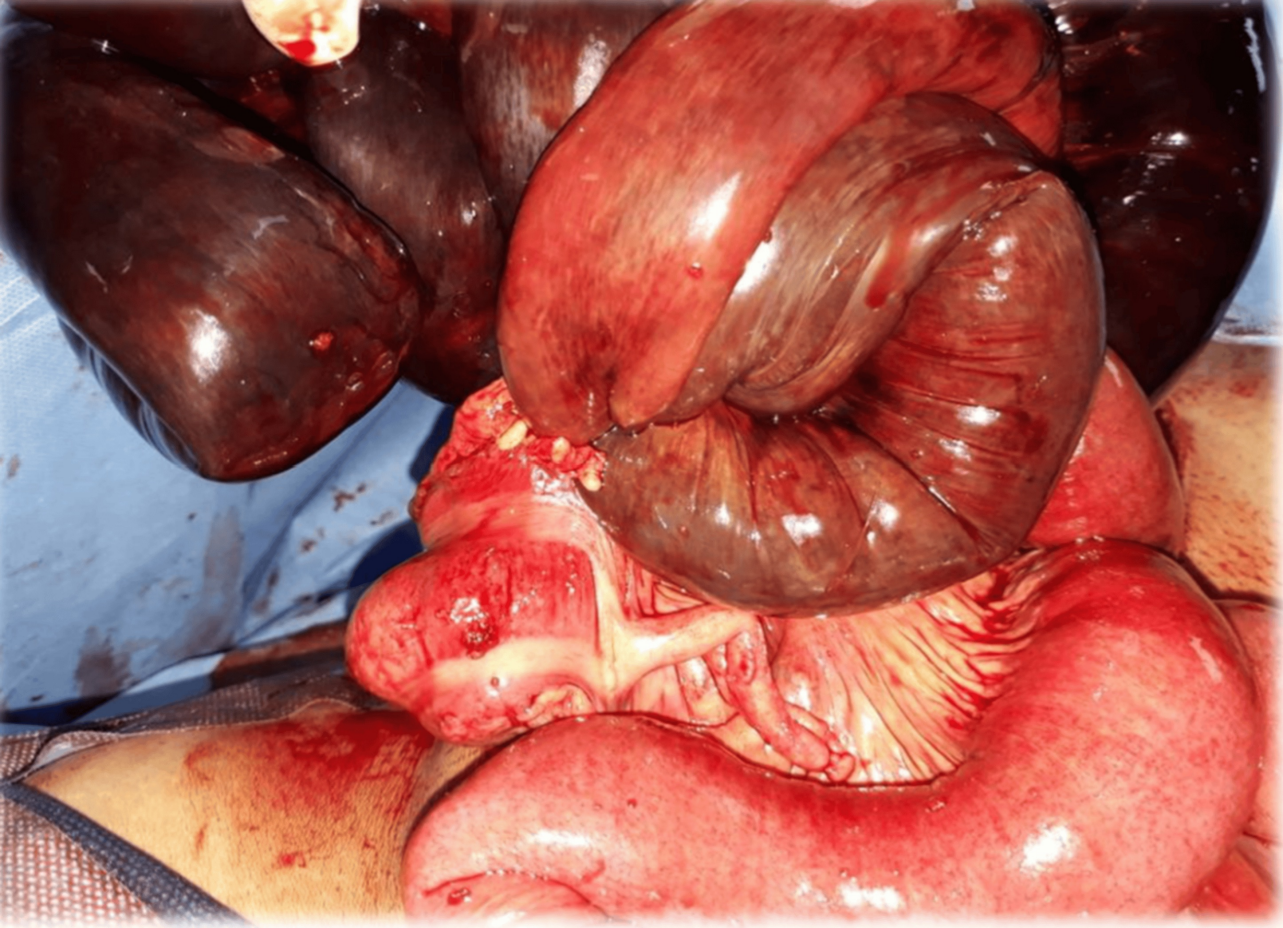
Case 1: Intraoperative image showing the knotting at the level of the terminal ileum near the cecum.

**Figure 4:**
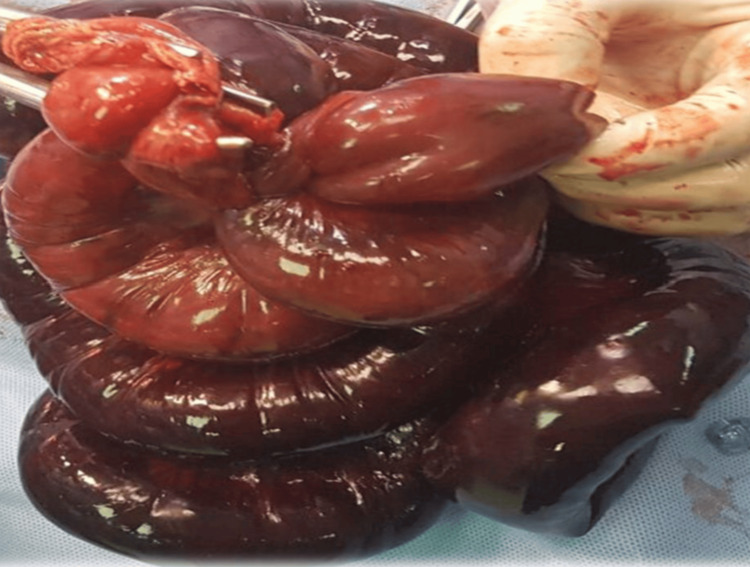
Case 1: En-bloc resection of the knotted small bowel segment.

Emergency laparotomy found hemoperitoneum and a knotted segment of small intestine (Figure 1). An additional intraoperative view of these intertwined loops is shown in Figure 2. The knot had caused gangrene of the ileal loops from the ileocecal region up to approximately 160 cm proximal to the ileocecal junction (Figure 3). The knot was not untangled due to extensive gangrene. A resection of the terminal ileum together with the cecum was performed as shown in Figure 4, and a side-to-side ileo-ascending colon anastomosis was constructed to restore bowel continuity.

Outcome

The patient’s postoperative course was uneventful. He recovered without complications and was discharged in stable condition.

Case 2

Case Presentation

A seven-year-old male with a history of splenectomy two years prior (post-traumatic) presented with clinical features of small bowel obstruction. He had no other significant medical history. Initially, he developed diffuse abdominal pain, vomiting, and abdominal distension, raising suspicion for adhesive small bowel obstruction secondary to prior surgery. The patient was admitted for conservative management, including bowel rest and nasogastric decompression. However, after 48 hours, his condition deteriorated acutely. He became tachycardic (heart rate ~170 bpm) and tachypnoeic (respiratory rate ~50 breaths/min), with new-onset abdominal guarding suggestive of strangulation and evolving peritonitis.

Diagnostic Workup

Arterial blood gas revealed metabolic acidosis (pH 7.27, HCO_3_ 9 mEq/L) and elevated lactate (8.6 mmol/L), indicative of systemic hypoperfusion and possible bowel ischemia. Laboratory investigations showed leucocytosis (WBC 19.8 ×10^3^/µL) and thrombocytosis (platelets 509 ×10^3^/µL). An abdominal X-ray demonstrated a nonspecific obstruction pattern, but given the rapid clinical decline, an emergent exploratory laparotomy was performed without further imaging.

Intraoperative Findings and Management

**Figure 5:**
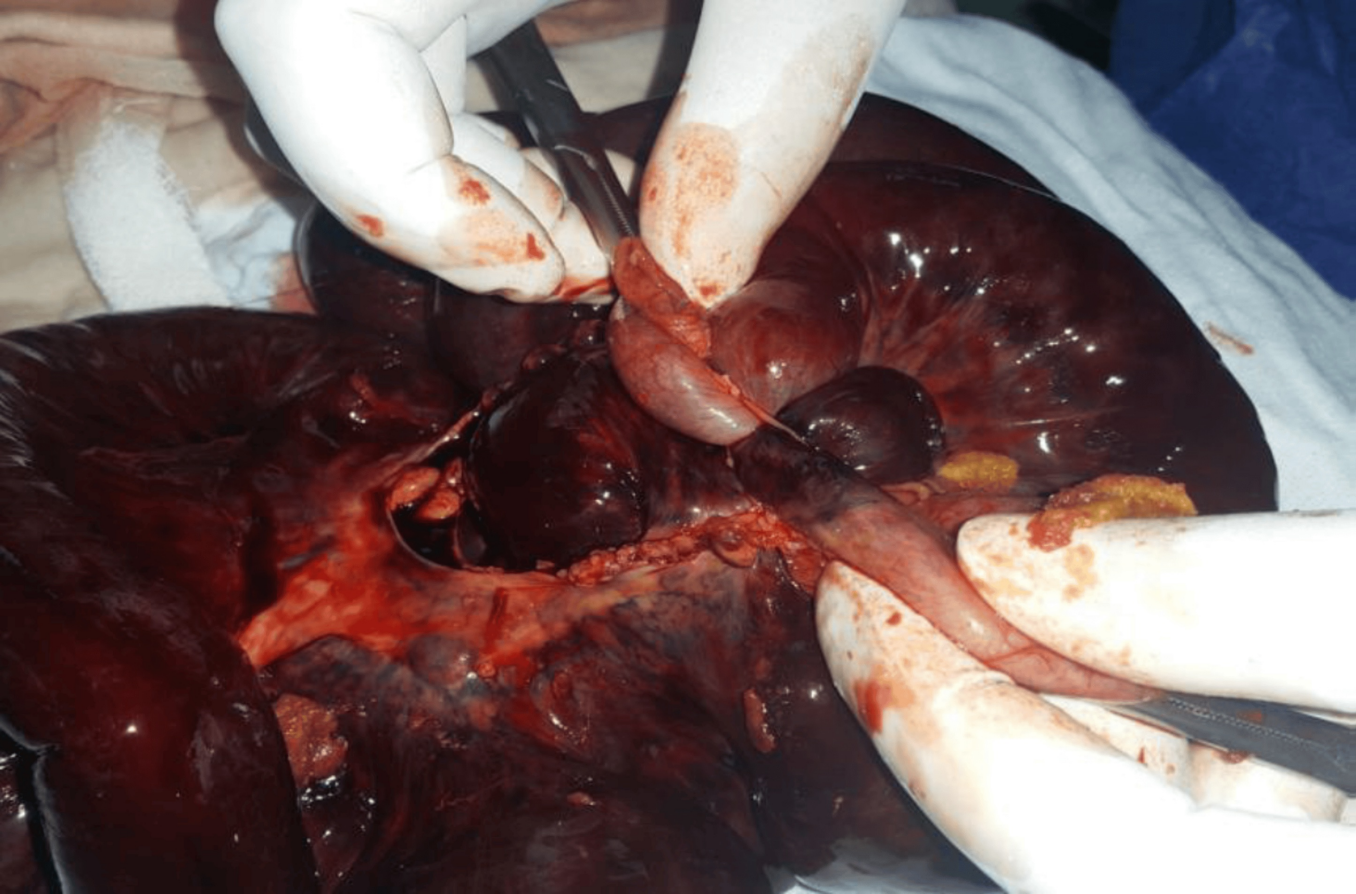
Case 2: Ileo-ileal knotting at the mid ileum showing gangrenous small bowel.

Surgical exploration revealed an adhesive band from the prior splenectomy, causing two ileal loops to form a tight knot, resulting in extensive small bowel strangulation as shown in Figure 5. Approximately 120 cm of ischemic ileum, from 150 cm distal to the duodenojejunal flexure to ~30 cm proximal to the ileocecal valve, was resected. The knotted segment was excised en bloc due to nonviability, and a primary end-to-end anastomosis was fashioned.

Outcome and Follow-Up

Postoperatively, the child was managed in the intensive care unit. His recovery was uneventful, with no anastomotic complications. After the return of bowel function, he was discharged home in good condition and remains well on follow-up.

Case 3

Case Presentation

A 45-year-old male with no prior surgical history presented to the emergency department with an acute onset of severe abdominal pain. Clinical examination revealed signs of generalized peritonitis, including diffuse tenderness with guarding, abdominal distension, and absent bowel sounds on auscultation. Despite these findings, his vital signs were initially stable, permitting further diagnostic imaging.

Diagnostic Workup

An upright abdominal X-ray revealed multiple air-fluid levels, suggestive of small bowel obstruction. Given his hemodynamic stability, a contrast-enhanced abdominal CT scan was performed. The CT demonstrated findings consistent with a closed-loop small bowel obstruction; however, a definitive “knot” was not clearly visualized. In view of possible strangulation on imaging and peritoneal signs on physical examination, an urgent exploratory laparotomy was undertaken.

Intraoperative Findings and Management

**Figure 6:**
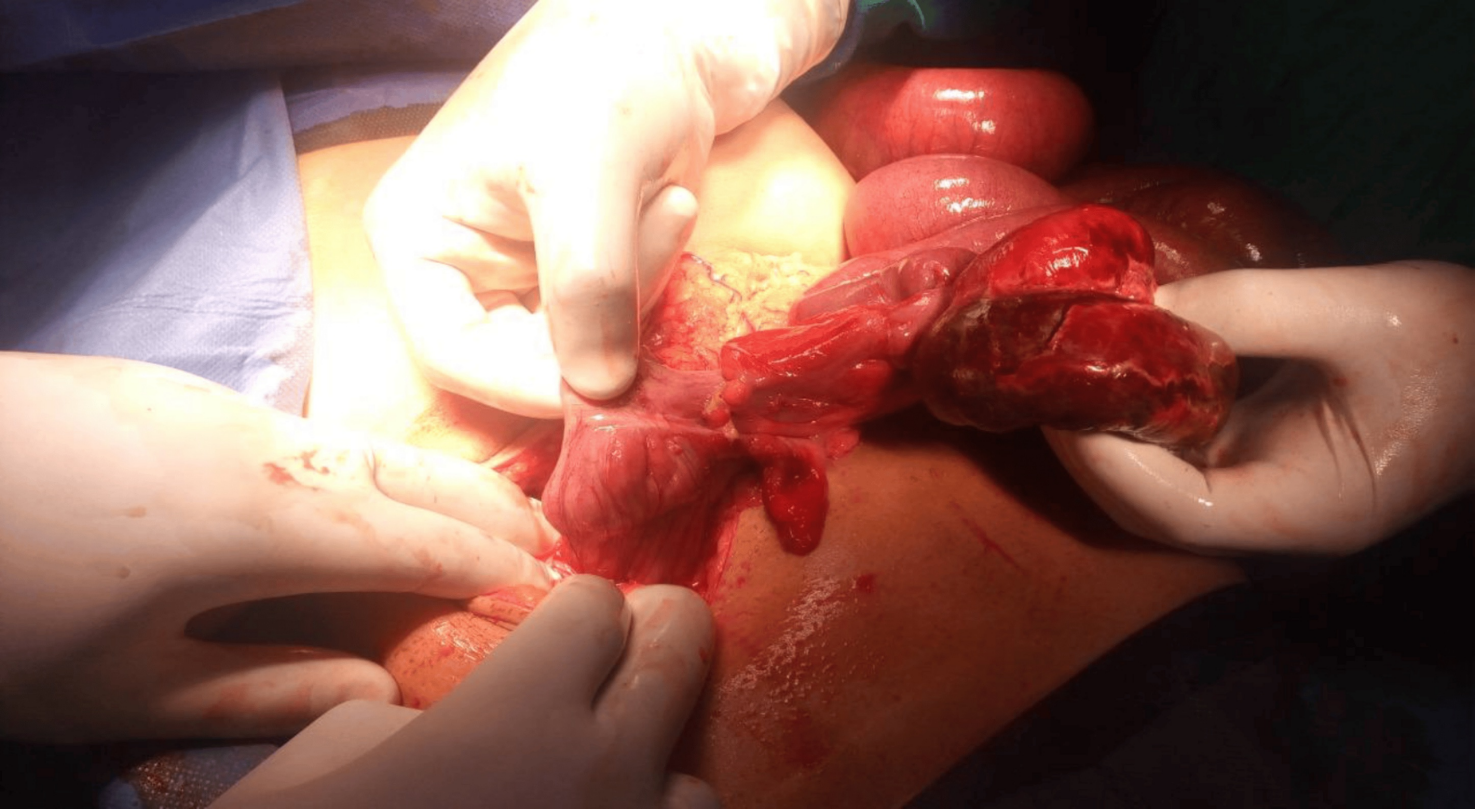
Case 3: Terminal ileal knotting and an inflamed appendix.

Surgical exploration identified an ileo-ileal knot involving the terminal ileum, as shown in Figure 6. Additionally, an inflamed appendix was found adjacent to the knotted loops. The compromised blood supply resulted in gangrene of approximately 20 cm of the terminal ileum. Given the proximity of the knot to the ileocecal region and involvement of the appendiceal base, a resection of the terminal ileum along with a right hemicolectomy (including the cecum and ascending colon) was performed. Bowel continuity was restored via a side-to-side ileotransverse anastomosis.

Outcome and Follow-Up

The postoperative course was uneventful. The patient tolerated oral intake by postoperative day 3 and was discharged on postoperative day 6 in stable condition. At follow-up, he remained asymptomatic with normal gastrointestinal function.

A detailed summary of the key clinical features, diagnostic workups, surgical interventions, and outcomes for each of the three cases is provided in Table 1.

**Table 1:** Key clinical features and management of the three cases. NG: nasogastric; ABG: arterial blood gas; CT: computed tomography

Clinical parameter	Case 1 (50 M)	Case 2 (7 M)	Case 3 (45 M)
Precipitating factor	None identified (no prior surgeries)	Adhesive band (post-splenectomy)	An inflamed appendicitis (lead point)
Presentation	Peritonitis with acute abdomen, melena, NG tube with coffee-ground aspirate (shock, HR 130)	Adhesive obstruction initially; later acute peritonitis with sepsis (tachycardia 170, metabolic acidosis)	Generalized peritonitis; vital signs initially stable
Diagnostic workup	X-ray: multiple air-fluid levels; no time for CT due to instability	X-ray: obstruction; ABG showing lactic acidosis; no time for CT (emergent surgery)	X-ray: obstruction; CT: closed-loop obstruction (preoperative)
Intraoperative findings	Ileo-ileal knot causing gangrene of the distal ileum and cecum (hemoperitoneum present)	Ileo-ileal knot induced by an adhesive band; extensive ischemia of the mid-ileum	Ileo-ileal knot of the terminal ileum with adjacent inflamed appendix; localized gangrene of the terminal ileum
Surgical management	Resection of the terminal ileum + cecum; side-to-side ileocolic anastomosis	Resection of ~120 cm of small bowel; primary end-to-end small bowel anastomosis	Resection of terminal ileum + right colon; ileotransverse (side-to-side) anastomosis
Outcome	Recovery without complications; discharged uneventfully	Recovery without complications; discharged after stabilization	Recovery without complications; discharged on postoperative day 6

## Discussion

Small bowel obstruction is one of the most common reasons for surgical ward admissions and represents a significant source of morbidity and healthcare expenditure worldwide. In the Western world, postoperative adhesions are the predominant cause of small bowel obstruction [1].

In developing countries such as Ethiopia, small bowel volvulus and abdominal wall hernias are the most common causes of small bowel obstruction. Other contributing factors include neoplastic lesions, ileosigmoid knotting, adhesions, intussusception, and the rare occurrence of ileo-ileal knotting. Effective management requires prompt fluid resuscitation and administration of antibiotics, particularly in patients with rapidly deteriorating clinical status who may necessitate emergency surgical intervention [4-7].

Ileo-ileal knotting is an exceptionally rare clinical condition characterized by a loop of proximal ileum encircling the distal ileum, resulting in a true knot. This phenomenon has been infrequently reported in the literature, both in developed and developing countries [8].

Published reports indicate gangrene rates as high as 78-80%, with a significant risk of morbidity and mortality associated with this condition [8,9]. The underlying cause of intestinal knotting, including ileo-ileal knotting, remains uncertain [10].

Preoperative diagnosis of ileo-ileal knotting remains highly challenging, as there are no pathognomonic etiological, clinical, or radiological features specific to this condition. Most cases are identified intraoperatively because patients typically present with features of intestinal obstruction that progress rapidly to ischemia and peritonitis. Previous studies have suggested that contrast-enhanced CT imaging may aid in the preoperative detection of intestinal knotting. Characteristic findings include the “whirl sign” produced by twisted bowel loops and sigmoid mesocolon, as well as a radial arrangement of intestinal loops and mesenteric vessels [11,12].

Management strategies depend on the viability of the affected bowel segment. When the bowel is viable, careful untying of the knot is recommended. However, in cases where the bowel is gangrenous, as in our patient, en bloc resection with either primary anastomosis or exteriorization is preferred to minimize the risk of perforation and further contamination [13].

When ileo-ileal knotting is suspected, prompt management is essential. Initial treatment involves aggressive intravenous fluid resuscitation, placement of a nasogastric tube for decompression, and administration of broad-spectrum antibiotics. Following adequate resuscitation, an emergency laparotomy should be performed. The surgical approach depends on the viability of the involved bowel segment. Some authors advocate untying the knot first to evaluate the extent of viable intestine and potentially minimize unnecessary bowel resection. Conversely, others caution against this strategy, citing concerns about contaminating the surgical field and the risk of releasing necrotic material into the circulation [14].

Recurrence of ileo-ileal knotting is rare, and if the bowel remains viable without evidence of strangulation, simple untwisting of the knot is generally recommended [13]. However, repeated attempts to release the knot carry a risk of perforation. In cases where the bowel is nonviable, en bloc resection of the gangrenous segment is preferred. This is typically performed after controlled decompression of the contents via enterotomy, followed by either exteriorization or primary anastomosis, depending on the surgeon’s judgment and the patient’s condition [13,15].

Postoperative management requires vigilant monitoring for hydration status, nutritional adequacy, electrolyte imbalances, anaemia, and early signs of anastomotic leakage if a primary anastomosis has been performed. In patients who have undergone extensive small bowel resection, follow-up should include assessment for short bowel syndrome and malnutrition. Should these complications arise, early intervention with dietary modifications and supportive therapy is essential to optimize outcomes. Additionally, preserving the ileocecal valve whenever possible is advisable, as it helps reduce the risk of short bowel syndrome by regulating intestinal transit and improving nutrient absorption [5].

## Conclusions

Ileo-ileal knotting is an exceptionally rare cause of acute abdominal catastrophe. This case series highlights that while the clinical presentation mimics common causes of bowel obstruction, a high index of suspicion is warranted in patients who deteriorate rapidly or have an unclear cause of obstruction. As preoperative diagnosis is often not possible, early exploratory surgery remains the cornerstone for diagnosis and treatment. Timely surgical intervention - with resection of nonviable bowel and appropriate anastomosis - can lead to excellent outcomes, as demonstrated by our patients. We advise clinicians to keep intestinal knotting in mind as a differential diagnosis of strangulated obstruction, albeit rare, as prompt treatment can be life-saving.
